# Characterization and expression analysis of Toll-interacting protein in common carp, *Cyprinus carpio* L., responding to bacterial and viral challenge

**DOI:** 10.1186/s40064-016-2293-3

**Published:** 2016-05-17

**Authors:** Shijuan Shan, Lei Wang, Fumiao Zhang, Yaoyao Zhu, Liguo An, Guiwen Yang

**Affiliations:** Shandong Provincial Key Laboratory of Animal Resistance Biology, College of Life Science, Shandong Normal University, Jinan, 250014 People’s Republic of China

**Keywords:** Tollip, Common carp (*Cyprinus carpio* L.), Molecular cloning, *Vibrio anguillarum*, Poly(I:C)

## Abstract

Toll-interacting protein (Tollip) is a mediator involved in the TLRs signaling pathway which is critical for innate immune response. In the present study, a full-length Tollip cDNA was first cloned from common carp (*Cc*Tollip), which was 1284 bp in length, containing an open reading frame of 831 bp encoding a peptide of 276 amino acids. Multiple sequence alignment showed that the *Cc*Tollip shared the highest similarity with that of grass carp and zebrafish. Phylogenetically, the *Cc*Tollip clustered together well with their piscine family members. Quantitative real-time PCR analysis indicated that *Cc*Tollip was widely expressed in all tissues tested and showed up-regulation with challenges of *Vibrio anguillarum* and poly(I:C), suggesting that *Cc*Tollip was activated by *V. anguillarum* and poly(I:C). These data indicated that *Cc*Tollip might play an important role in immune response to bacterial and viral invasion.

**Electronic supplementary material:**

The online version of this article (doi:10.1186/s40064-016-2293-3) contains supplementary material, which is available to authorized users.

## Background

Common carp (*Cyprinus carpio* L.) is an important aquaculture fish species worldwide, but many bacterial and viral infections have recently become a big problem in common carp aquaculture industry. A better understanding of the innate immune response in common carp against bacteria and virus is critical for the sustainable growth of the industry. In the innate immunity, the recognition of bacteria or virus by host cells is mediated by multiple pattern recognition receptors (PRRs), including Toll-like receptors (TLRs), NOD-like receptors (NLRs) and RIG-I-like receptors (RLRs). Among these PRRs, TLRs could recognize both bacteria and virus (Zhang and Gui 2012; Zhang et al. 2014).

Toll interacting protein (Tollip) is an important mediator in the innate immune responses induced by TLRs (Zhang and Ghosh 2002). It is first identified as a member of IL-1R pathway, which is presented in a complex with IRAK1 and inhibits IL-1-induced signaling by blocking IRAK1 phosphorylation (Burns et al. 2000). Because of the significant homology in the intracellular portion of TLRs and IL-1Rs, Tollip was also found to be involved in TLR-mediated signaling (Zhang and Ghosh 2002), which could associate directly with TLR2 or TLR4 and inhibit TLR-mediated cellular responses by suppressing phosphorylation and kinase activity of IRAK1. Tollip is the first direct substrate of IRAK1, and could be phosphorylated by activated IRAK1 upon stimulation with lipopolysaccharide (LPS) or IL-1 (Zhang and Ghosh 2002). Didierlaurent et al. reported that Tollip regulated the magnitude and kinetics of IL-6 and TNF-α production upon stimulation with IL-1β and low doses or physiological doses of LPS (Didierlaurent et al. 2006).

Besides the functions in mediating the innate immune responses, Tollip was also found to participate in protein sorting. Yamakami et al. described that the interaction of Tollip with Tom1, Ubiquitin and Clathrin in a high molecular mass complex involved in protein sorting (Yamakami et al. 2003; Yamakami and Yokosawa 2004). Katoh et al. suggested the similar result in their study, in which they found that Tollip and Tom1 formed a complex and regulated endosomal trafficking of ubiquitinated proteins (Katoh et al. 2004). Tollip is also involved in the nuclear translocation of proteins either as a sumoylation cofactor or a ligase (Ciarrocchi et al. 2009). Furthermore, Brissoni et al. clarified that Tollip is required in the sorting of the IL-1RI in the late endosomes (Brissoni et al. 2006). In addition, there is much information about the role of Tollip in some diseases nowadays (Liu et al. 2014a, b; Mukherjee and Biswas 2014; Shimizu et al. 2014). Tollip induces tolerance to the normal enteric flora by its up-regulated expression in intestinal epithelial cells (Melmed et al. 2003; Shibolet and Podolsky 2007). Maillard et al. reported that Tollip played an essential role on colitis susceptibility in mice (Maillard et al. 2014).

To date, the Tollip cDNA has been identified in many species including Atlantic salmon, rainbow trout, grass carp, grouper and Yesso scallop (Rebl et al. 2008; Huang et al. 2012; Li et al. 2015; Wei et al. 2015; Zhang et al. 2015). In these species, Tollip was found to participate in virus and bacteria induced immune responses. However, no information is known about the role of Tollip in innate immune response of common carp. In the present study, we first isolated and characterized the full-length cDNA of Tollip from common carp (named *Cc*Tollip), and examined the *Cc*Tollip mRNA expression patterns in various tissues under normal conditions. Furthermore, we analyzed the expression profile of the Tollip after stimulation with *Vibrio anguillarum*-one of the main fish pathogens and poly(I:C). These results implied that *Cc*Tollip might participate in the common carp immune response.

## Methods

### Fish rearing and tissue collection

Healthy common carp (*C. carpio* L.), with an average of 80 g, were collected from Fresh Water Fishery Research Institute of Shandong Province. The fish were cultured at 20 °C in circulating tap water and fed daily to satiation with commercial fish feed for more than 1 week prior to experimental use. Then the liver, spleen, gills, skin, muscle, head kidney, foregut, hindgut, buccal epithelium, brain and gonad were isolated for RNA extraction.

The protocol in this study was approved by the Ethics Committee on Animal Experiments of Medical School of Shandong University (Permit Number: ECAESDUSM 1420123009). All operations were performed under anesthesia, and all efforts were made to minimize suffering of the fish.

### RNA extraction and cDNA synthesis

Total RNA was isolated from various tissues, as mentioned above, using the RNAsimple Total RNA Kit (TIANGEN, China) according to the manufacturer’s protocols. The RNA template was reverse transcribed into first-strand cDNA by using FastQuant RT Kit (With gDNase) (TIANGEN, China) following the manufacturer’s instructions.

### Molecular cloning of Tollip gene

To clone Tollip gene from common carp, a pair of primers (Table 1) were designed based on the conserved region of reported Tollip sequences. The PCR template was synthesized by spleen-derived RNA of common carp. A 518 bp cDNA fragment of common carp Tollip was obtained by PCR using the cDNA templates. PCR program for amplification of common carp Tollip fragment was carried out under the following steps: initial denaturation was performed at 94 °C for 3 min, followed by 33 cycles at 94 °C for 30 s, 59 °C for 30 s, and 72 °C for 40 s, then a further 10 min extension step at 72 °C.

**Table 1:** Primers used in this study

Primer	Sequence (5′–3′)	Application	Annealing temperature (°C)
ToF	CTTGGGTATGCCGTCTAT	Amplification of Tollip cDNA	59
ToR	GTGCGAATCACCTCCTTA	Amplification of Tollip cDNA	59
GSP3′-1	CAGCCTGTGGTCCTGATG	3′RACE specific out primer	55
GSP3′-2	CCTGCTGTTACATCACAGA	3′RACE specific inner primer	55
GSP5′-1	GGTTCCCTCTCGCAGGCTCTCT	5′RACE specific out primer	55
GSP5′-2	ACGCCCTCTCATCGAAGATTTC	5′RACE specific inner primer	55
U3′-1	TACCGTCGTTCCACTAGTGATTT	3′RACE universal out primer	55
U3′-2	CGCGGATCCTCCACTAGTGATTTCACTATAGG	3′RACE universal inner primer	55
U5′-1	CATGGCTACATGCTGACAGCCTA	5′RACE universal out primer	55
U5′-2	CGCGGATCCACAGCCTACTGATGATCAGTCGATG	5′RACE universal inner primer	55
S11F	CCGTGGGTGACATCGTTACA	Real-time PCR for S11	60
S11R	TCAGGACATTGAACCTCACTGTCT	Real-time PCR for S11	60
RT-TF	CCGTCTCAGCATCACCGTCGTA	Real-time PCR for Tollip	60
RT-TR	TCGCTCCGTTGTGGGCAGTAG	Real-time PCR for Tollip	60
IL-1βF	GCTCGGCTTCATCTTGGAGAATGT	Real-time PCR for IL-1β	60
IL-1βR	GCAAGGTGAGGCTGGTCTTATTGT	Real-time PCR for IL-1β	60
IL-6F	TGAAGACAGTGATGGAGCAGCAGA	Real-time PCR for IL-6	60
IL-6R	CCTCACAGCAATGTGGCGAACA	Real-time PCR for IL-6	60

Then we obtained the full-length cDNA sequence of *Cc*Tollip using the RACE (Rapid amplification of cDNA ends). The 3′-RACE and 5′-RACE were performed using the 3′-full RACE core set (TaKaRa, Japan) and 5′-full RACE core set (TaKaRa, Japan) respectively following the manufacturer’s protocol. Two rounds of PCR were performed to amplify the 5′ and 3′ flanking regions, the primers and annealing temperatures were shown in Table 1.

All PCR fragments were purified by PCR purification kit (TIANGEN, China). The PCR products were cloned into the pMD18-T vector (TaKaRa, Japan) and then transformed into competent *Escherichia coli* DH5α cells. The selected single colonies were sequenced by BGI China. The 5′ and 3′ flanking regions were assembled with the known sequence.

### Sequence and bioinformatics analysis

The Tollip sequence was analyzed by BLAST program (http://blast.ncbi.nlm.nih.gov/Blast.cgi) at the NCBI server (www.ncbi.nlm.nih.gov/Structure/cdd/). The multiple sequence alignment of Tollip protein between common carp and other species was performed by Clustal W method. The conserved domain structures of Tollip were predicted by the SMART online software (http://smart.embl-heidelberg.de/). Phylogenetic tree was constructed based on the multiple sequence alignment with the full-length amino acid sequences of known Tollip using neighbor-joining method in MEGA 6.0 software program. Bootstrap sampling was reiterated 1000 times. The GenBank accession numbers or references for these sequences were shown in Additional file 1: Table S1.

### Immune challenges

The challenge experiments were divided into the bacterial (*V. anguillarum*) challenged group and the poly(I:C) challenged group.

For bacterial challenge, *V. anguillarum* (CCTCCM204067 strain) obtained from the China Center for Type Culture Collection, was incubated at 28 °C overnight in Luria–Bertani medium containing 3 % NaCl with shaking. The protocols were performed as previously described (Li et al. 2013, 2014; Yang et al. 2014). The *V. anguillarum* was inactivated in 0.5 % formalin at 4 °C overnight. The inactivated *V. anguillarum* was suspensed in sterile 0.1 M phosphate buffered saline (PBS) at 2 × 10^8^ CFU. The fish was challenged by intraperitoneal (i.p.) injection with 500 µl inactivated *V. anguillarum*. After challenge, all the fish were placed in a rectangular tank of fresh water. Sampling was performed 6, 12, 24 and 48 h after challenge, with three fish in each group, while fish in the control group were injected with the same amount of phosphate buffered saline (PBS).

Poly(I:C) (SIGMA, USA) dissolved in sterile PBS, was adjusted to 1.6 mg/ml. The fish was injected with poly(I:C) at 500 µl per fish by intraperitoneal (i.p.) injection. According to the results of our previous experiments, injection with PBS cannot up-regulate the expression of Tollip gene in common carp, and the expression level was similar to that in un-challenged fish. Thus, we used un-challenged fish as the control in the study. Seven immune-related tissues: liver, spleen, head kidney, hindgut, foregut, gills and skin were sampled and kept in liquid nitrogen for total RNA extraction.

### Isolation of head kidney leukocytes

Head kidney leucocytes (HKL) isolation and culture were performed based on the protocol described by Joerink et al. (2006). Head kidneys were aseptically excised and placed in a 100 m nylon cell strainer. Head kidneys were gently pressed with a plunger through a 100-m sterile nylon mesh and rinsed with PBS. Cell isolation was performed using a 51/34 % non-continuous percoll gradient (Sigma-Aldrich). After 25 min centrifugation at 800×*g*, the cells present in the interface of the gradient were collected and washed three times with PBS. The cells were resuspended in complete L-15 (Gibco) (supplemented with 5 % FBS, 100 U/ml penicillin, and 100 µg/ml streptomycin). About 10^7^ cells/well were seeded in 24-well plate with 500 µl complete mediums. After recovering overnight at 25 °C, drug treatment was performed using lipopolysaccharide (LPS) (10 µg/ml, Sigma-Aldrich). Then the cells were collected at 3, 6, 9, 12 and 24 h. Total RNA was isolated from the HKLs and qPCR was performed with the gene-specific primers (Table 1) to evaluate the mRNA levels of Tollip, IL-1β, and IL-6.

### Quantitative real-time PCR

Real-time PCR was performed in triplicate for each sample on an iQ5 Real-time PCR instrument (Bio-Rad) using SYBR Green Real Master Mix (TIANGEN, China). 40S ribosomal protein S11 gene served as an internal reference gene to normalize the mRNA expression in different tissues as previous studies described (Engelsma et al. 2001). The primers that were used have been shown in Table 1. Reaction conditions were as follows: incubated for 1 min at 94 °C, followed by 40 cycles of 20 s at 94 °C, 20 s at 59 °C and 50 s at 70 °C. The PCR data were analyzed using 2^(−ΔΔCt)^ method.

### Statistical analysis

Standard deviations were calculated using the relative expression ratios of 3 replicates for each gene measured. Data were analyzed with the Graphpad Prism 5 software, with two-way analysis of variance (ANOVA), and statistical significance was defined as *p* < 0.05.

## Results

### Cloning and sequence characterization of *Cc*Tollip

**Fig. 1:**
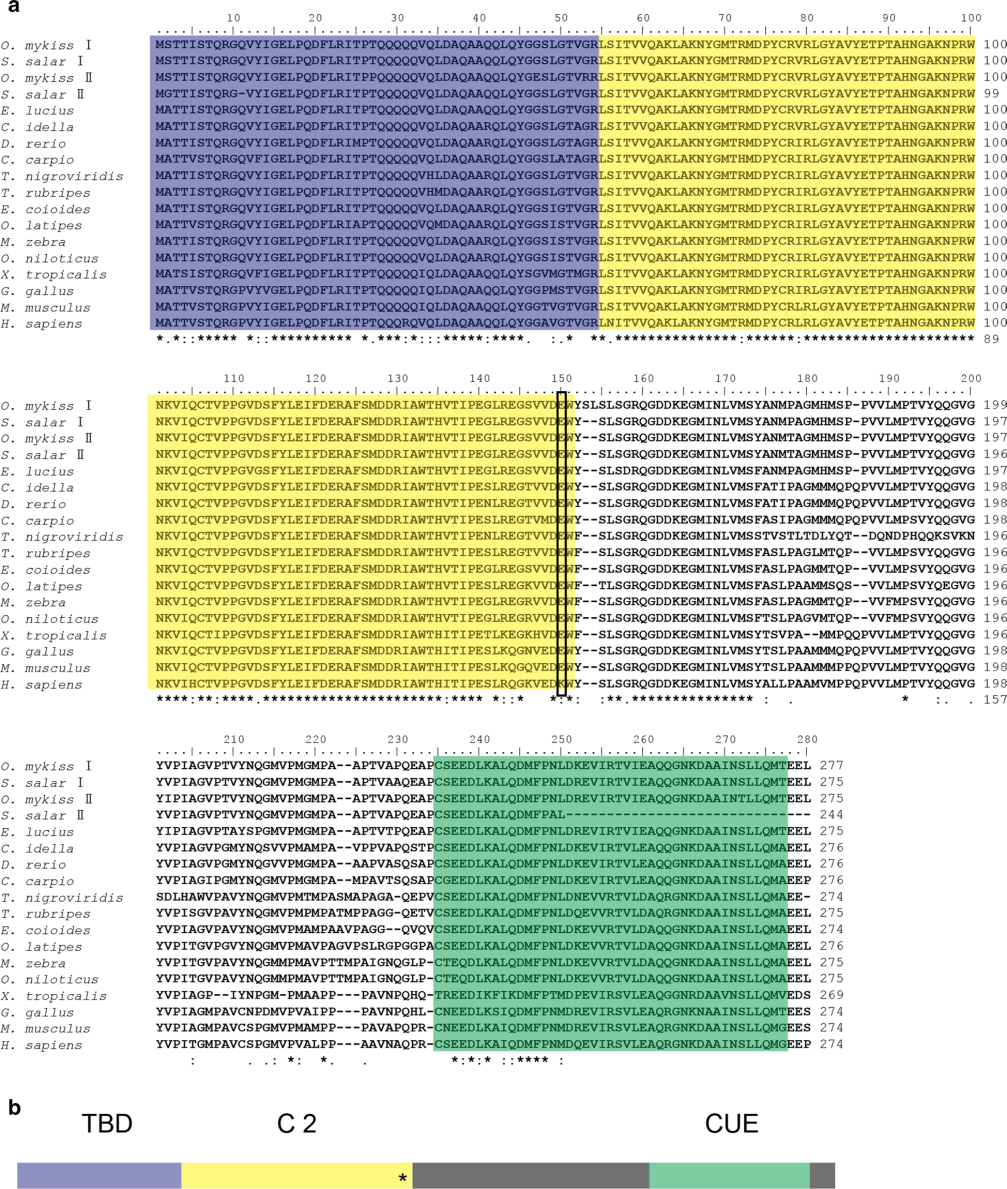
Multiple alignment and schematic representation of Tollip. **a** Amino acid sequence alignment and functional domains of Tollip. The sequences were aligned by ClustalX 2.0. The identical amino acid residue is indicated in the *asterisk* (*); the predicted domains from SMART Server software have been indicated by *colored boxes*: the *blue* denotes the TBD (Tom1-binding domain), the *yellow* shows the C2 (conserved core 2) domain, the *green* indicates the CUE (coupling of ubiquitin to endoplasmic reticulum degradation) domain. The *black box* points the amino acid at position 150. The GenBank accession numbers of these sequences are listed in Additional file 1: Table S1. **b** Schematic representation of the structural domains of Tollip. The predicted domains from SMART Server software are indicated by *colored boxes* as shown in Fig. 1a, while other regions are *shaded* in *grey color*. The *asterisk* (*) represent the amino acid at the position 150

The full-length cDNA of *Cc*Tollip was 1284 bp containing 92 bp 5′-untranslated region (UTR), an open reading frame (ORF) of 831 bp encoding a peptide of 276 amino acids and a 361 bp 3′-UTR. The poly (A) tail was at 361 bp downstream of stop codon TAG. The polyadenylation signal ATTAAA was located 20 bp upstream of the poly (A) tail (Additional file 2: Fig. S1). The Tollip sequence was deposited in GenBank under accession no. KF660221. Conserved domain analysis with SMART program showed that *Cc*Tollip contained a TBD (Tom1-binding domain) extending from Met-1 to Arg-54, a C2 (conserved core domain 2) domain extending from Leu-55 to Trp-151, and a CUE (coupling of ubiquitin to endoplasmic reticulum degradation) domain extending from Cys-231 to Ala-273 (Fig. 1).

### Alignment and phylogenetic analysis

**Fig. 2:**
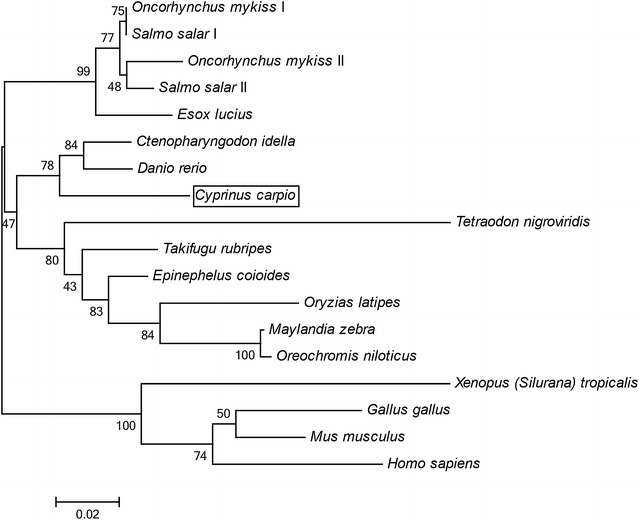
Phylogenetic tree of Tollip amino acid sequences. The phylogenetic tree was produced by neighbor-joining method in MEGA 6.0. The numbers at tree nodes indicate the boot-strap percentage of 1000 bootstrap samples. The frame shows the common carp Tollip reported in the study. The GeneBank accession numbers of these sequences are listed in Additional file 1: Table S1

The deduced amino acid sequences of Tollip were compared between common carp and other species (Additional file 3: Table S2). The *Cc*Tollip shared the highest similarity with the Tollip of zebrafish (93.8 %) and grass carp (93.1 %), whereas a low similarity to *Xenopus tropicalis* (79.9 %). The multiple sequence alignment of Tollip between common carp and the other species showed that the sequences of TBD, C2 domain and the CUE domain remained more conserved than the other regions (Fig. 1). To further investigate the evolutionary relationship of Tollip, the phylogenetic tree was generated, which showed that the *Cc*Tollip was clustered with other bony fish Tollip and it was most closely related to the grass carp and zebrafish Tollip (Fig. 2), suggesting that the direction of Tollip protein evolution was consistent with the evolution of species.

### Expression profile of *Cc*Tollip

**Fig. 3:**
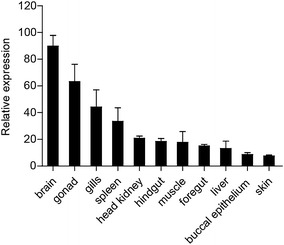
Tissue expression of *Cc*Tollip in normal common carp. The *Cc*Tollip transcripts in the spleen, head kidney, foregut, hindgut, skin, gills, buccal epithelium, liver, muscle, gonad and brain of the common carp were detected by real-time PCR. 40S ribosomal protein S11 in each tissue was amplified as internal control

In order to analyze the *Cc*Tollip expression profile, the relative expression of *Cc*Tollip gene was detected in 11 tissues of healthy common carp (Fig. 3). The results showed that the *Cc*Tollip was predominantly detected in brain, followed by gonad, gills, spleen, head kidney, hindgut, muscle, foregut and liver, and the lowest expression levels were found in buccal epithelium and skin.

### The temporal expression of *Cc*Tollip after stimulation with *V. anguillarum*

**Fig. 4:**
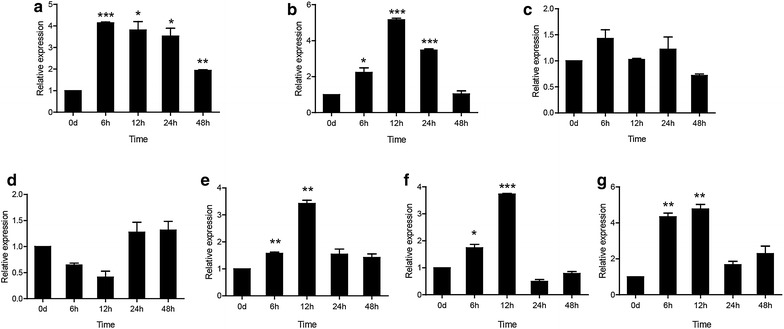
The relative expression levels of *Cc*Tollip in various tissues of common carp after i.p. injection with *V. anguillarum*. Relative expression of *Cc*Tollip in the liver (**a**), spleen (**b**), head kidney (**c**), foregut (**d**), hindgut (**e**), gills (**f**) and skin (**g**) of the common carp at different time points is shown; these results were calculated relative to the expression of the 40S ribosomal protein S11 gene. All samples were analyzed in triplicate. *Each bar* represents the mean ± SD, n = 3; *p < 0.05, **p < 0.01 and ***p < 0.001 versus the control group (denoted by 0 h). The experiments were repeated three times

**Fig. 5:**
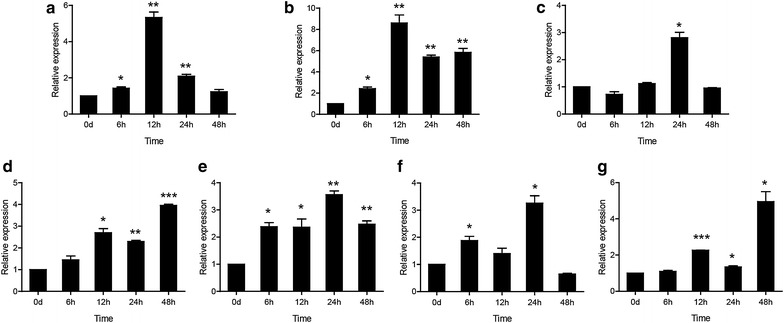
The relative expression levels of *Cc*Tollip in various tissues of common carp after i.p. injection with poly(I:C). Relative expression of *Cc*Tollip in the liver (**a**), spleen (**b**), head kidney (**c**), foregut (**d**), hindgut (**e**), gills (**f**) and skin (**g**) of the common carp at different time points is shown; these results were calculated relative to the expression of the 40S ribosomal protein S11 gene. All samples were analyzed in triplicate. *Each bar* represents the mean ± SD, n = 3; *p < 0.05, **p < 0.01 and ***p < 0.001 versus the control group (denoted by 0 h). The experiments were repeated three times

To investigate the potential role of *Cc*Tollip in response to bacterial and viral infections, the expression profile of *Cc*Tollip in various tissues after *V. anguillarum* (Fig. 4) and poly(I:C) stimulation (Fig. 5) was determined.

After stimulus with *V. anguillarum*, the expression of *Cc*Tollip gene in liver, spleen, hindgut, skin and gills was up-regulated, and then gradually decreased over time. The expression level of *Cc*Tollip in liver was induced and reached the peak at 6 h (about 4.15-fold; *p* < 0.001) (Fig. 4a). In spleen, hindgut, gills and skin, the level of Tollip transcripts started to increase at 6 h and reached the maximum at 12 h (5.16-fold, 3.42-fold and 3.73-fold, 4.77-fold respectively; *p* < 0.001 or *p* < 0.01) (Fig. 4b, e–g). However, the expression level of Tollip in head kidney and foregut had no change after stimulation with *V. anguillarum* (Fig. 4c, d).

### The temporal expression of *Cc*Tollip after stimulation with poly(I:C)

After challenge with poly(I:C), the *Cc*Tollip gene expression in liver and spleen was up-regulated and reached to peak at 12 h (5.33-fold and 8.61-fold, *p* < 0.01) (Fig. 5a, b). In head kidney, hindgut and gills, the peak of Tollip gene expression was observed at 24 h after challenge, which were 2.81-fold, 3.56-fold and 3.26-fold (*p* < 0.05 or *p* < 0.01), respectively (Fig. 5c, e, f). The highest induction level of Tollip in foregut and skin was both at 48 h after challenge, 3.95-fold and 4.95-fold (*p* < 0.001 or *p* < 0.05) were observed, respectively (Fig. 5d, g).

### Expression profile of CcTollip, IL-1β and IL-6 in HKLs

**Fig. 6:**
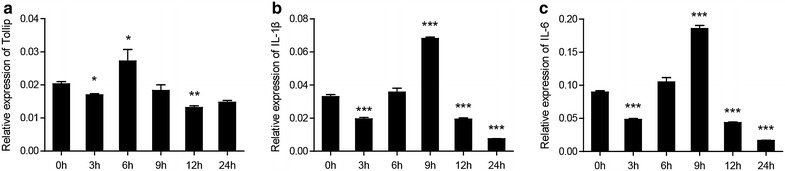
Expression patterns of *Cc*Tollip, IL-1β and IL-6 gene induced by LPS in HKLs. **a** Induction expression of *Cc*Tollip by LPS. **b** Induction expression of IL-1β by LPS. **c** Induction expression of IL-6 by LPS. Three groups of HKLs were treated with LPS for 3, 6, 9, 12 and 24 h. Error bars represent the mean ± SD obtained by measuring each sample three times from three independent experiments. The shown data have been normalized to 40S ribosomal protein s11 gene. The experiments were repeated three times

In order to study the correlation of Tollip and proinflammatory cytokines, we isolated the head kidney leukocytes. It was found that the expression of Tollip, IL-1β and IL-6 presents fluctuation after LPS stimulation. The expression of Tollip arrived at the highest value at 6 h, with a 1.4-fold of the control group (*p* < 0.05) (Fig. 6a). While the IL-1β and IL-6 mRNA expression reached the highest level at 9 h, which were 2.06-fold and 2.07-fold of the control group, respectively (*p* < 0.001) (Fig. 6a, b).

## Discussion

Tollip is an important regulator in TLRs mediating innate immune response. In this study, Tollip was isolated from common carp, and the role of *Cc*Tollip was investigated after stimulation with *V. anguillarum* and poly(I:C).

Structural analysis revealed that *Cc*Tollip contained a conserved TBD, C2 and CUE domain. Tollip can interact with Tom1 and participate in the ubiquitination pathway through the TBD and CUE domain (Yamakami et al. 2003). *Cc*Tollip shared the same domains with other species, which suggested that *Cc*Tollip played the similar role as the other species Tollip did. Li et al. reported that overexpression of Tollip inhibited NF-κB reporter gene transcription in human monocytic THP-1 cells, and a point mutation of the lysine residue at position 150 to glutamic acid (K150E) could abolish this function of Tollip (Li et al. 2004). Although in fish and some other species, Tollip has a glutamic acid residue at this position. Rebl et al. reported that overexpression of the trout Tollip in HEK-293 cells also reduced TLR-mediated NF-κB activation (Rebl et al. 2011), suggesting that the C2 domain is essential in the function of Tollip to inhibit the activation of NF-κB pathway, although C2 domain in fish Tollip has different key amino acid residue compared with human.

From the multiple sequence alignment, the *Cc*Tollip shared the highest similarity with the Tollip of zebrafish (93.8 %) and grass carp (93.1 %) (Additional file 3: Table S2). Phylogenetic tree showed that the *Cc*Tollip was clustered with other bony fish Tollip and it was most closely related to the grass carp and zebrafish Tollip (Fig. 2). Furthermore, in the tree, the Tollip of salmonid fish divided into Tollip I and Tollip II, while the common carp only had one kind of Tollip, which was similar to other cyprinid fish.

The Tollip expression analysis has been investigated in several species, it is found that Tollip is ubiquitously expressed in many tissues or organs (Sandor and Buc 2005; Gunthner et al. 2013; Nishimura and Naito 2005; Lv et al. 2012; Rebl et al. 2008; Li et al. 2015). In this study, *Cc*Tollip was constitutively expressed in all tissues tested and it was highly expressed in brain of common carp. The previous studies have shown that interleukin-1 receptor type I (IL-1RI) and recruiting a signaling core complex consisting of the myeloid differentiation primary response protein 88 (MyD88) are differently distributed in the hippocampus and in the subcellular compartments of primary hippocampal neurons, suggesting that the members of IL-1R cascade participate in the neuronal signaling pathway (Gardoni et al. 2011). Tollip as a member of IL-1R signaling pathway may have a high expression in brain. The distribution of grouper Tollip confirmed our observation (Wei et al. 2015).

Tollip was reported to have the negatively regulatory function in mammalian (Burns et al. 2000; Rebl et al. 2008), while its expression was up-regulated in viral infected trout (Rebl et al. 2008) and grass carp (Huang et al. 2012), which relates to that the up-regulation of Tollip in fish may counteract the infection-related pro-inflammatory activation of the innate immune system and protect against pathological effects (Rebl et al. 2010). In the present study, we found that the expression of *Cc*Tollip was also up-regulated in many tissues of common carp after bacteria and poly(I:C) challenge. Spleen is one of the most important immune organs, in which there are plenty of lymphocytes, granulocytes and monocytes (Chen et al. 2013; Lieschke and Trede 2009). In this study, after injection with *V. anguillarum* and poly(I:C), the change of *Cc*Tollip expression level in the spleen of common carp was highest, which indicated that Tollip might play an important immune role in spleen.

The liver is the main source of acute-phase proteins (Baumann and Gauldie 1994; Nazemi et al. 2014) and has a unique vascular system receiving the majority of the blood supply faces continuous exposure to foreign pathogens and commensal bacterial products which could trigger TLRs signaling (Nakamoto and Kanai 2014). At the same time, the occurring of anti-inflammatory response could avoid the injury of liver (Nakamoto and Kanai 2014). Thus, the expression of *Cc*Tollip in liver of common carp was significantly up-regulated after pathogen infection. However, the expression level of Tollip in head kidney and foregut had no change after stimulation with *V. anguillarum*. The previous study in *Aeromonas salmonicida* Infection Rainbow Trout confirmed the pronounced expression of *Cc*Tollip in liver and the unchanged expression in head kidney (Brietzke et al. 2015). Furthermore, we examined the expression of Tollip and proinflammatory cytokines. It was found that the mRNA level of Tollip, IL-1β, and IL-6 was up-regulated in HKLs, suggesting that there may be a correlation between Tollip and proinflammatory cytokines. The mechanism of the relationship between Tollip and proinflammatory cytokines in common carp need to be further investigated.

Tollip was reported to play an important role in epithelium-mediated cytolysis in pathogen clearance (Huang et al. 2012). And increased Tollip concentration was found in human intestinal cells, which was hypothesized to be a result of continuous exposure to the intestinal microflora (Melmed et al. 2003; Cario and Podolsky 2005). So the *Cc*Tollip transcript was significantly up-regulated in gut, skin and gills, which were the sites of potential pathogen entry.

Interestingly, different from the tissues above, the expression level of *Cc*Tollip in head kidney and foregut had no change after infection with *V. anguillarum* (Fig. 4c, d). Thus, *Cc*Tollip may have multiple functions in common carp, and further studies on the function of *Cc*Tollip need to be done.

## Conclusion

In this study, we described the identification of *Cc*Tollip and the results suggested that *Cc*Tollip might play a role in the immune system of common carp, which established a basis for further examination of the *Cc*Tollip function. However, further investigation is needed to explore the antibacterial and antiviral mechanisms, which might be helpful in the development of new methods for preventing infections in common carp.

## Additional files

10.1186/s40064-016-2293-3 GeneBank accession numbers of Tollip used in this study.
10.1186/s40064-016-2293-3 Nucleotide and deduced amino acid sequences of *Cc*Tollip. The nucleotide sequence, which encodes amino acid of Tollip is shown by capital, and amino acid sequence is shown below the nucleotide sequence. 5′- and 3′-untranslated region are shown in lower case. The putative polyadenylation site (attaaa) is shown in bold.
10.1186/s40064-016-2293-3 Percent identity of Tollip between common carp and other species.

## Abbreviations

Tollip: Toll interacting protein
*V. anguillarum*: *Vibrio anguillarum*
TLR: Toll-like receptor
PRR: pattern recognition receptor
NLR: NOD-like receptor
RLR: RIG-I-like receptor
RACE: rapid amplification of cDNA ends
ORF: open reading frame
TBD: Tom1-binding domain
C2: conserved core domain 2
CUE: coupling of ubiquitin to endoplasmic reticulum degradation
