# Supplementary material for: Characterization and expression analysis of Toll-interacting protein in common carp, Cyprinus carpio L., responding to bacterial and viral challenge
Source: Springerplus. 2016 May 17;5:639. doi: 10.1186/s40064-016-2293-3 (PMC4870529; doi:10.1186/s40064-016-2293-3)
Supplement: Supplementary file 1 — 10.1186/s40064-016-2293-3 GeneBank accession numbers of Tollip used in this study. [file 40064_2016_2293_MOESM1_ESM.docx]

**Table S1** GeneBank accession numbers of Tollip used in this study

| Species | Protein | GeneBank accession no. |
| --- | --- | --- |
| *Homo sapiens*  *Mus musculus*  *Salmo salar*  *Salmo salar*  *Oncorhynchus mykiss*  *Oncorhynchus mykiss*  *Ctenopharyngodon idella*  *Danio rerio*  *Cyprinus carpio*  *Tetraodon nigroviridis*  *Gallus gallus*  *Maylandia zebra*  *Oryzias latipes*  *Takifugu rubripes*  *Oreochromis niloticus*  *Esox lucius*  *Xenopus (Silurana) tropicalis* | Tollip  Tollip  Tollip I  Tollip II  Tollip I  Tollip II  Tollip  Tollip  Tollip  Tollip  Tollip  Tollip  Tollip  Tollip  Tollip  Tollip  Tollip | EAX02433  AAH62139  CAM88653, CAM88654  CAM88655, CAM88656  CAI48085  CAI48086  AFM09715  AAH46009  AHB53117  CAF93239  NP_001006471  XP_004568758  XP_004084776  XP_003978697  XP_003459909  ACO14514  AAH76673 |
