# Supplementary figures and images for: Characterization and expression analysis of Toll-interacting protein in common carp, Cyprinus carpio L., responding to bacterial and viral challenge
Source: Springerplus. 2016 May 17;5:639. doi: 10.1186/s40064-016-2293-3 (PMC4870529; doi:10.1186/s40064-016-2293-3)

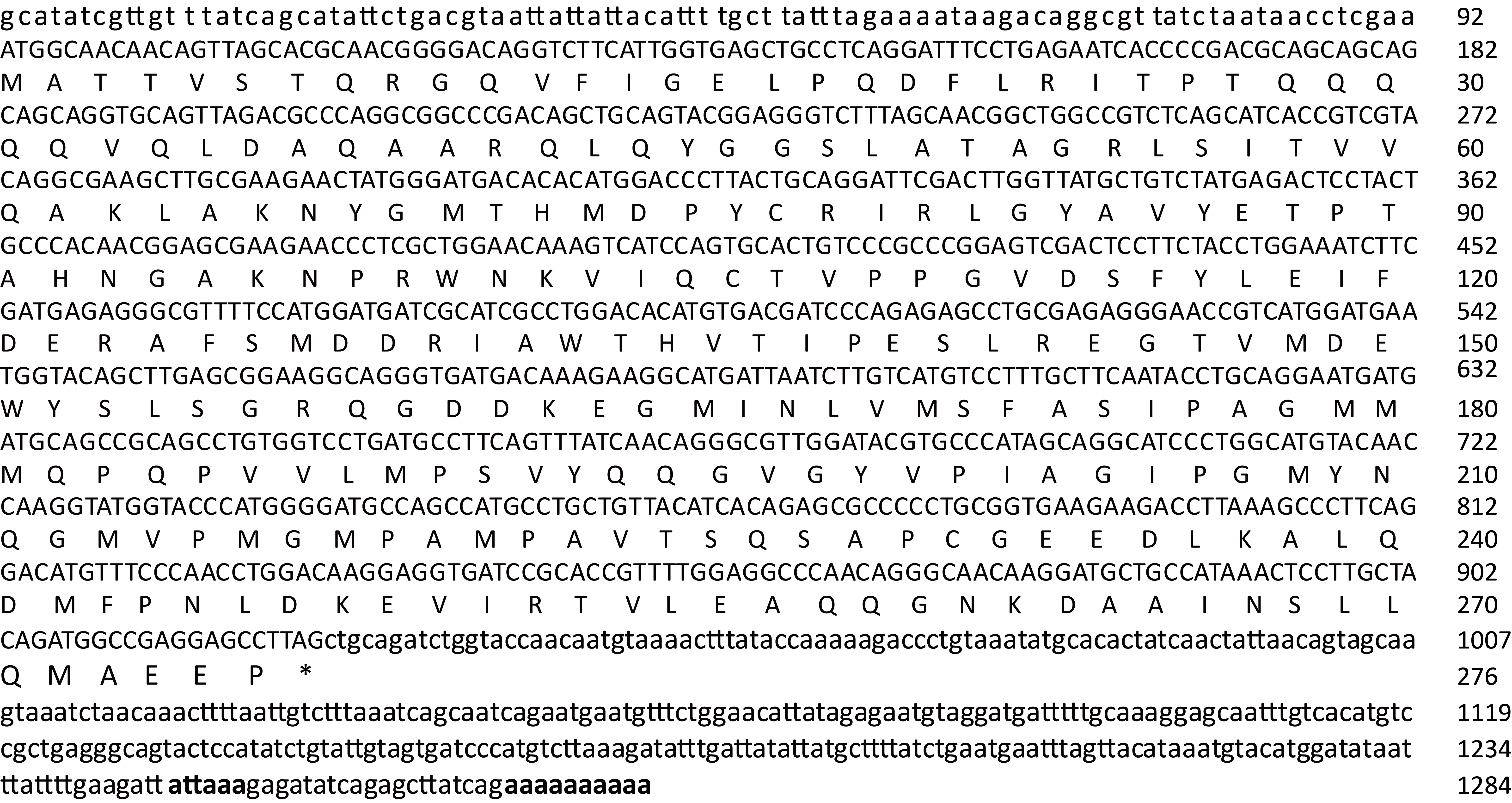

Supplement: Supplementary file 2 — 10.1186/s40064-016-2293-3 Nucleotide and deduced amino acid sequences of CcTollip. The nucleotide sequence, which encodes amino acid of Tollip is shown by capital, and amino acid sequence is shown below the nucleotide sequence. 5′- and 3′-untranslated region are shown in lower case. The putative polyadenylation site (attaaa) is shown in bold. [file 40064_2016_2293_MOESM2_ESM.tif]
