# Supplementary material for: Characterization and expression analysis of Toll-interacting protein in common carp, Cyprinus carpio L., responding to bacterial and viral challenge
Source: Springerplus. 2016 May 17;5:639. doi: 10.1186/s40064-016-2293-3 (PMC4870529; doi:10.1186/s40064-016-2293-3)
Supplement: Supplementary file 3 — 10.1186/s40064-016-2293-3 Percent identity of Tollip between common carp and other species. [file 40064_2016_2293_MOESM3_ESM.docx]

**Table S2** Percent identity of Tollip between common carp and other species

| Species | Percent identity |
| --- | --- |
| *Oncorhynchus mykiss* I  *Salmo salar* I  *Oncorhynchus mykiss* II  *Salmo salar* II  *Esox lucius*  *Ctenopharyngodon idella*  *Danio rerio*  *Tetraodon nigroviridis*  *Takifugu rubripes*  *Epinephelus coioides*  *Oryzias latipes*  *Maylandia zebra*  *Oreochromis niloticus*  *Xenopus tropicalis*  *Gallus gallus*  *Mus musculus*  *Homo sapiens* | 89.5  89.1  86.9  88.5  88.4  93.1  93.8  80.2  88.3  89.4  87.2  87.2  86.4  79.9  82.5  83.6  81.8 |
